# Pregnancy Protects against Abnormal Gut Permeability Promoted via the Consumption of a High-Fat Diet in Mice

**DOI:** 10.3390/nu15245041

**Published:** 2023-12-08

**Authors:** Caio F. Biolcatti, Vanessa C. Bobbo, Carina Solon, Joseane Morari, Roberta Haddad-Tovolli, Eliana P. Araujo, Marcela R. Simoes, Licio A. Velloso

**Affiliations:** 1 Laboratory of Cell Signaling, Obesity and Comorbidities Research Center, University of Campinas, Campinas 13083-864, Brazil; caiobiolcatti@gmail.com (C.F.B.); vanessa.bobbo@yahoo.com.br (V.C.B.); cssolon@gmail.com (C.S.); morarij@gmail.com (J.M.); haddad@recerca.clinic.cat (R.H.-T.); earaujo@unicamp.br (E.P.A.); marcela.rsimoes@gmail.com (M.R.S.); 2 School of Medical Sciences, University of Campinas, Campinas 13083-894, Brazil; 3 School of Nursing, University of Campinas, Campinas 13083-887, Brazil; 4 Neuronal Control of Metabolism (NeuCoMe) Laboratory, Institut d’Investigacions Biomèdiques August Pi i Sunyer (IDIBAPS), 08036 Barcelona, Spain

**Keywords:** obesity, reproduction, metabolism, fatty acid, microbiota

## Abstract

The consumption of large amounts of dietary fats and pregnancy are independent factors that can promote changes in gut permeability and the gut microbiome landscape. However, there is limited evidence regarding the impact of pregnancy on the regulation of such parameters in females fed a high-fat diet. Here, gut permeability and microbiome landscape were evaluated in a mouse model of diet-induced obesity in pregnancy. The results show that pregnancy protected against the harmful effects of the consumption of a high-fat diet as a disruptor of gut permeability; thus, there was a two-fold reduction in FITC–dextran passage to the bloodstream compared to non-pregnant mice fed a high-fat diet (*p* < 0.01). This was accompanied by an increased expression of gut barrier-related transcripts, particularly in the ileum. In addition, the beneficial effect of pregnancy on female mice fed the high-fat diet was accompanied by a reduced presence of bacteria belonging to the genus *Clostridia*, and by increased *Lactobacillus murinus* in the gut (*p* < 0.05). Thus, this study advances the understanding of how pregnancy can act during a short window of time, protecting against the harmful effects of the consumption of a high-fat diet by promoting an increased expression of transcripts encoding proteins involved in the regulation of gut permeability, particularly in the ileum, and promoting changes in the gut microbiome.

## 1. Introduction

Maternal obesity is an important risk factor for the early development of metabolic diseases in offspring [1]. In humans, the increased risk for offspring health disorders associated with maternal obesity was described for the first time in 1949, in a study that evaluated 5000 pregnant women [2]. Thereafter, several studies strengthened this concept, demonstrating that these health problems are not restricted to childhood but extend to the entire lifespan, increasing the risk for obesity, hypertension, type 2 diabetes (DM2), and several other medical conditions that are currently regarded as obesity comorbidities [3,4,5]. Experimental studies have mostly reproduced findings in humans, and the optimization of animal models for studying the impact of gestational obesity on progeny has leveraged the progress in the field [6].

Maternal obesity impacts offspring health via distinct mechanisms, such as disturbing placenta physiology [7], altering milk composition [8], promoting epigenetic changes in gene expression and function [9], affecting the development of brain circuits involved in the regulation of energy balance [10], and impairing the development of the blood-brain barrier [11]. Over the last decade, studies have identified changes in the gut microbiota as yet another important mechanism driving the harmful effects of gestational obesity upon offspring health [12,13]. Both the consumption of a high-fat diet (HFD) and pregnancy can act independently or in combination to modify the mother’s gut microbiota landscape and affect offspring health [14,15]. Based on studies that evaluated obese non-pregnant humans and animal models, it has been shown that drivers of systemic metabolism could be affected by changes in gut permeability resulting from dysbiosis [16,17]. However, it is currently uncertain if the consumption of a HFD during pregnancy has a similar effect on gut dysbiosis and permeability as the one observed in the non-pregnant condition [18,19].

Here, gut microbiota and markers of intestinal permeability were evaluated in non-pregnant and pregnant mice fed either a standard diet (chow) or a HFD. To our surprise, pregnancy protected against the defective gut permeability promoted by the consumption of a HFD. This was accompanied by the increased ileal expression of transcripts encoding for proteins involved in gut barrier integrity and by the reduced presence of bacteria belonging to the *Clostridia* genus in the gut.

## 2. Materials and Methods

### 2.1. Mice

**Figure 1:**
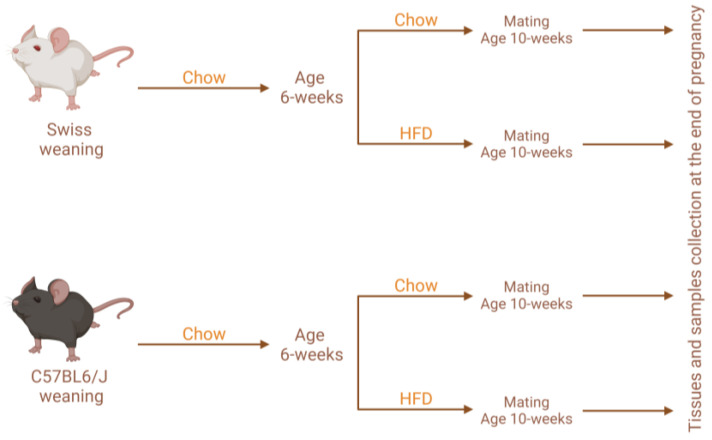
Experimental protocol design. HFD, high-fat diet.

Swiss and C57BL6/J (C57) mice were obtained from the Multidisciplinary Center for Biological Investigation on Laboratory Animal Science at the University of Campinas (Campinas, São Paulo, Brazil) (protocol numbers: 3934-1 and 5133-1) and were housed at 22 °C (+/−1 °C) with a 12:12 h light/dark cycle and ad libitum access to water and food. The models used in this study were previously published elsewhere [6,7]. Once the animals reached 6 weeks of age, the females were separated into two groups: (1) a control group, which received a chow diet (Nuvilab, Nuvilab, Colombo, Brazil; 3.85 kcal/g; 19.2 g/100 g protein, 67.3 g/100 g carbohydrate, and 4.3 g/100 g fat) (Table 1), and (2) an intervention group, which was fed a HFD (5.4 kcal/g; 24 g/100 g protein, 26 g/100 g carbohydrate, and 41 g/100 g fat) (Table 1). After four weeks on either diet, half of the females of each group were mated with male mice; from that point on, female mice were placed into individual cages. Therefore, for each mouse strain (C57 and Swiss), there were four groups under study: (1) non-pregnant (NP) chow (Swiss *n* = 5; C57 *n* = 10), (2) pregnant (P) chow (Swiss *n* = 5; C57 *n* = 12), (3) non-pregnant HFD (Swiss *n* = 5; C57 *n* = 10), and (4) pregnant HFD (Swiss *n* = 5; C57 *n* = 11). When mice reached 2 weeks of pregnancy (therefore, 12 weeks of life), we performed euthanasia and collected their feces (Swiss mice) or their duodenum, jejunum, ileum, and colon (C57 mice). Figure 1 depicts the experimental protocol.

### 2.2. Mating

Female mice were exposed to males for 24 h in an enriched environment (mixed shavings and plastic tubes). After one week, due to weight variations (using a high-precision scale, twice a week), it was possible to detect which females were pregnant. When females completed two weeks of pregnancy, along with their counterparts, they were euthanized.

### 2.3. Microbiota Analysis

Feces were collected at the time of defecation (*n* = 5/group), directly into clean tubes, to prevent environmental contamination. Then, they were immediately placed in liquid nitrogen and were stored at −80 °C. Subsequently, samples were sent on dry ice to Neoprospecta Microbiome Technologies (Florianópolis, Santa Catarina, Brazil), which performed the complete sequencing of the bacterial DNA present in the fecal material.

### 2.4. RNA Extraction and Quantitative Real-Time PCR

After euthanasia and tissue extractions, samples (*n* = 5–6/group) were stored at −80 °C in a bio-freezer until utilization. RNA extraction was performed using Trizol reagent (Applied Biosystems, Foster City, CA, USA) following manufacturer instructions. A high-capacity cDNA Reverse Transcription kit (Applied Biosystems, Foster City, CA, USA) was used for cDNA synthesis. The quantification of genes was performed via the StepOne Real-Time PCR system (Applied Biosystems) using LuminoCt qPCR ReadyMix (Cat No. L6669, Sigma-Aldrich, St. Louis, MO, USA). For the duodenum, Rpl0 was used as a housekeeping gene. The averages between GAPDH, β-actin and Rpl0 were used as housekeeping genes for the colon, jejunum, and ileum, as individually, these genes were modulated between the different intervention groups. The complete list of primers is specified in Table 2.

### 2.5. Immunohistochemistry and Image Analysis

Intestinal specimens—the duodenum, jejunum, ileum, and colon—were collected (*n* = 5–6/group) and embedded in a buffer solution containing 10% paraformaldehyde for fixation. After fixation, tissues were dehydrated in ethanol, embedded in paraffin, and stained with hematoxylin and eosin for histological analysis (H&E, Merck, Rahway, NJ, USA). After preparing the microscope slides, specimens were analyzed and photographed using a light microscope.

### 2.6. Quantitative Analysis of Gut Permeability

Quantitative analysis of gut permeability was performed via the serum quantification of oral fluorescent sugar (*n* = 3–4/group). When female mice, both pregnant and non-pregnant, and on a HFD or chow diet, had completed 12 weeks, they were submitted to five hours of fasting. After fasting, the mice received an aqueous solution, via gavage, containing sugar labeled with fluorescein isothiocyanate (FITC-Dextran, Merck, USA). Four hours after administration, the mice were anesthetized, and blood was collected via intracardiac punction. Blood was centrifuged at 3000× *g* and 4 °C, for 10 min, and plasma was extracted and diluted. This was used for the fluorescence reading of FITC-Dextran, and the results were compared to a control curve with known values of FITC-Dextran.

### 2.7. Statistical Analysis

All values were expressed as means ± standard error of the mean (SEM). A comparison between the four groups (two-factors) was achieved using a two-way ANOVA followed by Sidak’s or Tukey’s multiple comparison tests. Non-pregnant chow was considered the control and *p* < 0.05 was defined as a significant difference.

## 3. Results

### 3.1. Pregnancy Protects against Diet-Induced Increase in Gut Permeability

**Figure 2:**
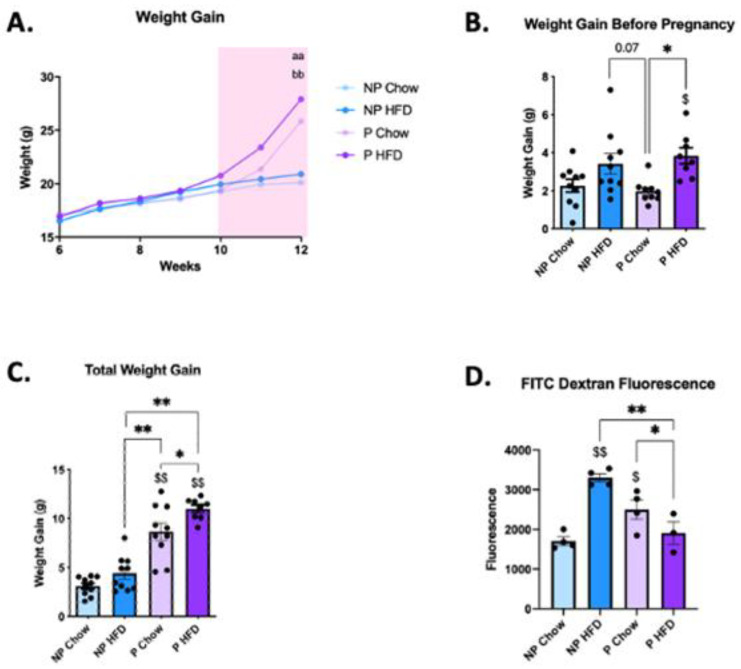
Pregnancy protection against diet-induced defective gut permeability, in C57 female mice. (**A**) Weight change throughout the total duration of the experiment; (**B**) weight gain before pregnancy; (**C**) total weight gain (prior to the mating and during pregnancy); (**D**) gut permeability measured via FITC dextran fluorescence. In (**A**–**C**), *n* = 8–10; in (**D**), *n* = 3–4. In (**A**), aa *p* < 0.01 NP chow vs. P chow, and bb *p* < 0.01 NP HFD vs. P HFD; in (**B**–**D**), $ *p* < 0.05 and $$ *p* < 0.01 vs. control (NP chow); * *p* < 0.05 and ** *p* < 0.01 to compare groups except NP chow. if not shown, n.s.

Body mass variation during the whole experimental period is depicted in Figure 2A. During the four-week period prior to mating, female mice on a HFD gained more body mass than females fed chow (Figure 2B) did. This was also true during the pregnancy period, as females on a HFD gained more body mass than females fed chow (Figure 2C). In non-pregnant females, the consumption of the HFD promoted a 2-fold increase in gut permeability (Figure 2D). However, to our surprise, in pregnant females on a HFD, gut permeability remained similar to the control levels (Figure 2D).

### 3.2. Consumption of a High-Fat Diet in Pregnant Mice Promotes Minimal Changes in the Duodenum

**Figure 3:**
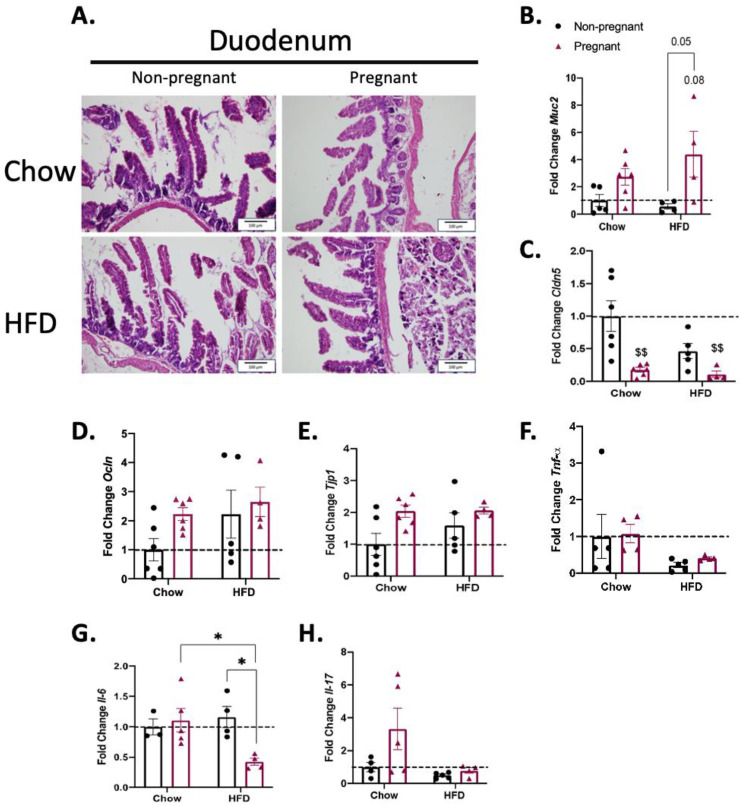
Effect of pregnancy in the duodenum of mice. (**A**) Representative histological images of the duodenum of mice. Left images are for non-pregnant mice; right images are for pregnant mice; top images are for mice on a chow diet; bottom images are for mice on a HFD; scale = 100 μm; (**B**) mRNA quantification, expressed as fold change (NP chow as control) of *Muc2*; (**C**) *Cldn5*; (**D**) *Ocln*; (**E**) *Tjp1*; (**F**) *Tnf-α*; (**G**) *Il-6*; and (**H**) *Il-17*. (**A**) A representative image, *n* = 5–6; in (**B**–**E**), *n* = 4–6; in F, *n* = 4–5; in G, *n* = 3–5; in H, *n* = 4–5. In (**B**–**H**), $$ *p* < 0.01 vs. control (NP chow); * *p* < 0.05 to compare groups except NP chow; if not shown, n.s.

To explore the mechanisms behind the protective effect of pregnancy on diet-induced defective gut permeability, mice were submitted to the evaluation of each portion of the intestine. In the duodenum, neither the consumption of the HFD nor pregnancy promoted major changes on microscopic architecture (Figure 3A). In addition, both conditions promoted only minor changes in the expression of transcripts encoding for proteins involved in the gut permeability barrier (Figure 3B–E), and in transcripts encoding for inflammatory proteins (Figure 3F,G). Thus, pregnancy was accompanied by the increased expression of *Muc2* in female mice on a HFD (Figure 3B), a reduction in *Cldn5* in female mice on both chow and a HFD (Figure 3C), and a reduction in *Il-6* in female mice on a HFD (Figure 3G).

### 3.3. Consumption of a High-Fat Diet in Pregnant Mice Promotes Minimal Changes in the Jejunum

**Figure 4:**
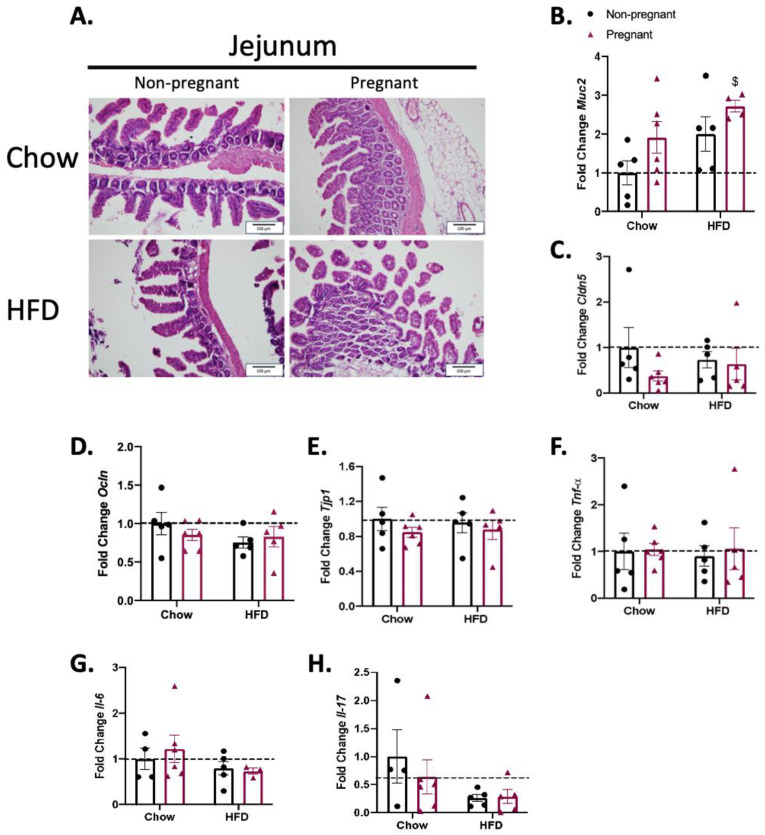
Effect of pregnancy in the jejunum of mice. (**A**) Representative histological images of the jejunum of mice. Left images are for non-pregnant mice; right images are for pregnant mice; top images are for mice on a chow diet; bottom images are for mice on a HFD; scale = 100 μm; (**B**) mRNA quantification, expressed as fold change (NP chow as control) of *Muc2*; (**C**) *Cldn5*; (**D**) *Ocln*; (**E**) *Tjp1*; (**F**) *Tnf-α*; (**G**) *Il-6*; and (**H**) *Il-17*. (**A**) A representative image with *n* = 5–6; (**B**) *n* = 4–6; (**C**–**F**) *n* = 5–6; (**G**) *n* = 3–6; (**H**) *n* = 4–6. (**B**–**H**) $ *p* < 0.05 vs. control (NP chow); if not shown, n.s.

As for the duodenum, neither the consumption of the HFD nor pregnancy promoted major changes in the microscopic architecture of the jejunum (Figure 4A). The evaluation of the expression of transcripts encoding for proteins involved in the gut permeability barrier (Figure 4B–E) and inflammatory proteins (Figure 4F–H) revealed an increase in the expression of *Muc2* in pregnant mice on a HFD only (Figure 4B).

### 3.4. Pregnancy Promotes the Increased Expression of Gut Permeability-Related Transcripts in the Ileum of Mice on a High-Fat Diet

**Figure 5:**
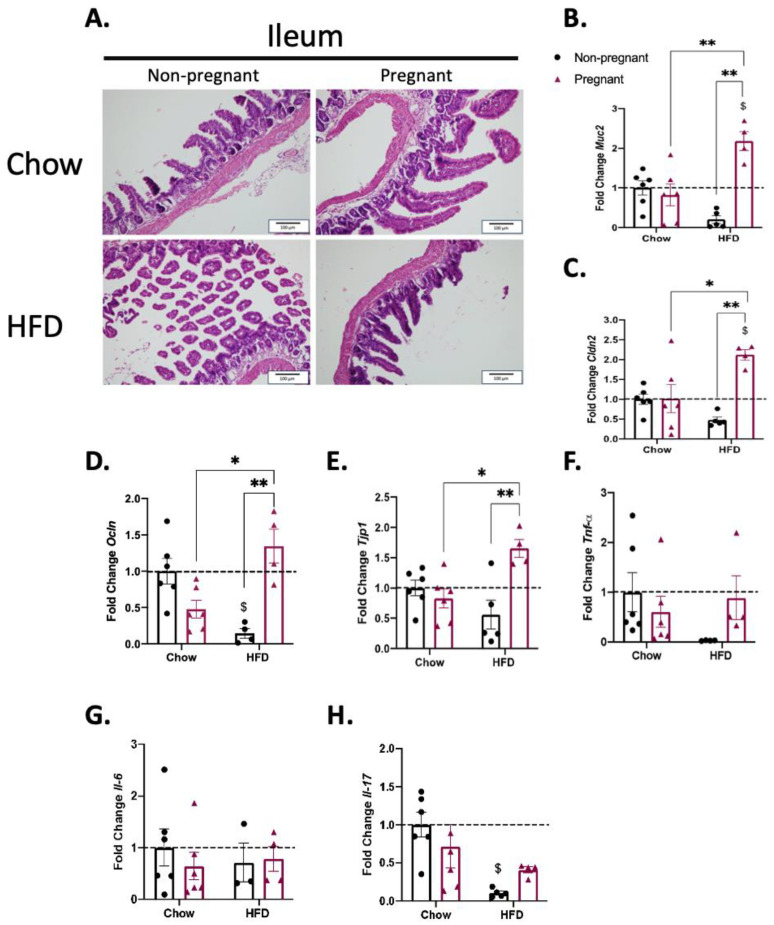
Effect of pregnancy in the ileum of mice. (**A**) Representative histological images of the ileum of mice. Left images are for non-pregnant mice; right images are for pregnant mice; top images are for mice on a chow diet; bottom images are for mice on a HFD; scale = 100 μm; (**B**) mRNA quantification, expressed as fold change (NP chow as control) of *Muc2*; (**C**) *Cldn2*; (**D**) *Ocln*; (**E**) *Tjp1***;** (**F**) *Tnf-α*; (**G**) *Il-6*; and (**H**) *Il-17.* (**A**) Representative image with *n* = 5–6; (**B**–**G**), *n* = 3–6; H, *n* = 4–6. (**B**–**H**) $ *p* < 0.05 vs. control (NP chow); * *p* < 0.05 and ** *p* < 0.01 to compare groups except NP chow; if not shown, n.s.

The microscopic analysis revealed that a HFD and/or pregnancy did not promote major microscopic structural changes in the ileum of mice (Figure 5A). However, the determination of ileum transcript expression revealed increases in *Muc2* (Figure 5B), *Ocln* (Figure 5D), and *Tjp1* (Figure 5E) in pregnant mice on a HFD. This was accompanied by no changes in the transcripts encoding for inflammatory proteins (Figure 5F–H).

### 3.5. Consumption of a High-Fat Diet in Pregnant Mice Promotes Minimal Changes in the Colon

**Figure 6:**
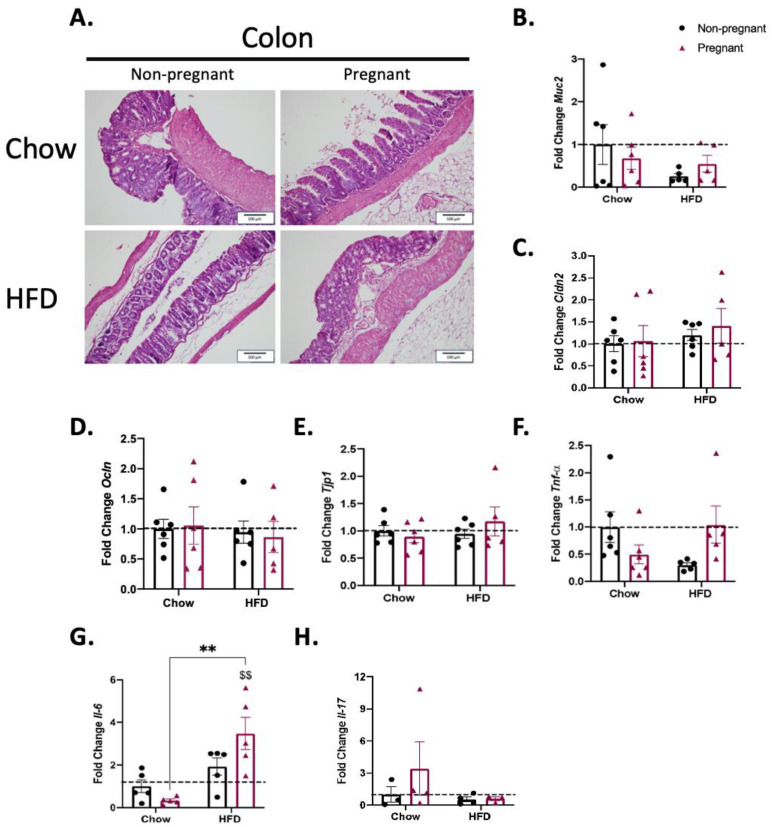
Effect of pregnancy in the colon of mice. (**A**) Representative histological images of the colon of mice. Left images are for non-pregnant mice; right images are for pregnant mice; top images are for mice on a chow diet; bottom images are for mice on a HFD; scale = 100 μm; (**B**) mRNA quantification, expressed as fold change (NP chow as control) of *Muc2*; (**C**) *Cldn2*; (**D**) *Ocln*; (**E**) *Tjp1***;** (**F**) *Tnf-α*; (**G**) *Il-6*; (**H**) *Il-17.* (**A**) Representative image with *n* = 5–6; in (**B**–**F**) *n* = 5–6; (**G**) *n* = 5; H *n* = 3–4. (**B**–**H**) $$ *p* < 0.01 vs. control (NP chow); ** *p* < 0.01 to compare groups except NP chow; if not shown, n.s.

HFD and/or pregnancy promoted no major microscopic structural changes in the colon of mice (Figure 6A). In addition, there were no changes in the expression of transcripts encoding for proteins involved in the gut permeability barrier (Figure 6B–E). Regarding the expression of transcripts encoding for inflammatory proteins (Figure 6F–H), there was only an effect of diet when increasing the expression of *Il-6* in the colon of pregnant mice (Figure 6G).

### 3.6. Increased Lactobacillus Murinus and Reduced Bacteria of the Genus Clostridia in the Gut of Pregnant Mice Fed a High-Fat Diet

**Figure 7:**
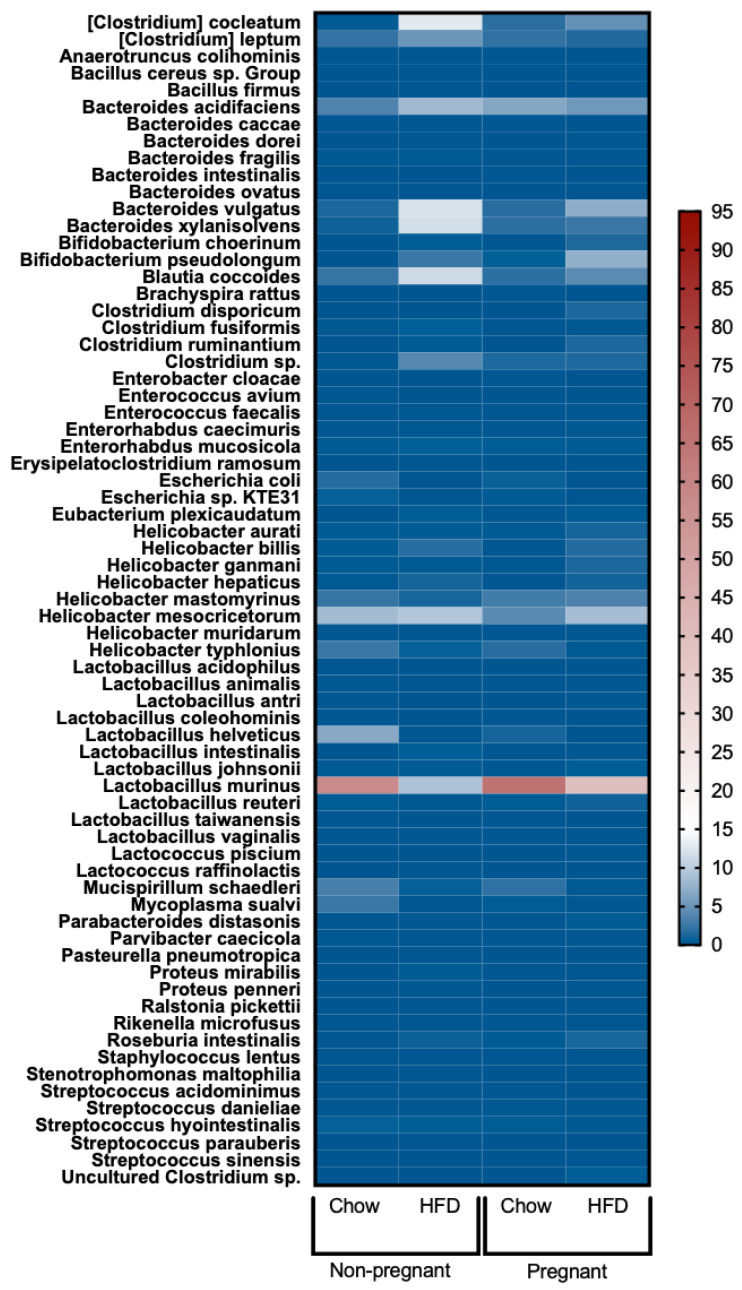
Heatmap of gut microbiota in female mice. From left to right: non-pregnant chow, non-pregnant HFD, pregnant chow, and pregnant HFD. In all groups, *n* = 4–5.

As changes in gut barrier permeability and the consumption of large amounts of dietary fats are frequently accompanied by changes in the gut microbiome landscape, this study investigated whether the beneficial changes promoted by pregnancy could be accompanied by changes in the gut microbiome. The results revealed that, as compared to non-pregnant mice on a HFD, pregnancy in mice on a HFD was accompanied, predominantly, by an increase in *Lactobacillus murinus* and by reductions in the presence of bacteria of the *Clostridia* genus (Figure 7).

## 4. Discussion

In this study, it was shown that pregnancy protects against diet-induced damage to gut barrier permeability by promoting an increased expression of transcripts encoding for proteins involved in gut barrier integrity, particularly in the ileum, and this was accompanied by increased presence of *Lactobacillus murinus* and a reduced presence of bacteria of the genus *Clostridia* in the gut.

Prior studies have described in detail the effects of a HFD on gut barrier integrity and also on the gut microbiome landscape [16,20,21]. In addition, there are some studies that have evaluated the same parameters in pregnancy [12,22]. However, little was known about the impact of pregnancy on gut permeability and microbiota in diet-induced obesity [2,13,19]. This scientific question has great epidemiological implications because of the continuous increase in the worldwide prevalence of obesity (www.worldobesity.org), and particularly because the number of women with obesity entering pregnancy is also increasing [23,24].

In the first part of the study, the model was validated showing that female mice on a HFD for four weeks prior to pregnancy presented increased body mass compared to that of the control, and that there was a match between the group selected for pregnancy and the group selected for non-pregnancy. In addition, during pregnancy, the group that received a HFD maintained a higher body mass compared to the pregnant mice fed chow. Thus, the model reproduced findings reported previously by others [6].

Next, using the FITC-dextran method, it was shown that, as expected, in non-pregnant mice on a HFD, there was a 2-fold increase in gut permeability, whereas in pregnant mice fed chow, there was a 1.5-fold increase in gut permeability, and these findings mostly reproduce those of previous studies [12,16,20,21,22]. Conversely, pregnancy completely abolished the harmful effect of the HFD on gut barrier integrity, and, to our knowledge, this has never been described before.

As in several conditions affecting the gut barrier, structural changes may occur in certain segments of the intestine [25,26,27]. Gut morphology was evaluated via conventional microscopy in hematoxylin-/eosin-stained sections. As previously described in similar experimental models, there were no major differences in any of the gut segments evaluated, which reinforced the finding that our model matches the ones of prior studies [28,29].

Gut barrier integrity is maintained through a combination of factors, such as the following: the mucus layer, which provides a physical and chemical barrier separating the components of the gut lumen and the mucosal cells [30]; the tight junctions and adherence complexes composed of claudins, E-cadherin, occludin and other junctional adhesion molecules that provide a tight link between epithelial cells [31]; the components of the mucosal innate immune system [32]; and sophisticated cell-autonomous immunity mechanisms in the mucosal epithelial cells [33]. Here, we evaluated components of three of these mechanisms: a transcript encoding for a protein of the mucus layer, transcripts encoding for proteins of tight junctions and adherence complexes, and transcripts encoding for cytokines of mucosal innate immunity.

Regarding the innate immune response, there were no major changes in the expression of cytokines throughout the distinct segments of the gut in either HFD-fed and/or pregnant mice. The only minor changes occurred in the duodenum, where the combination of a HFD and pregnancy was accompanied by the reduced expression of *Il-6*, and in the colon, where the combination of a HFD and pregnancy was accompanied by the increased expression of *Il-6*. In other models of diet-induced obesity in the absence of pregnancy, studies have shown heterogeneous results. In mice fed a diet rich in refined carbohydrates there was only a minor increase in gamma-interferon, with no changes in *Tnfa* [34]; in another study, mice fed a HFD for 8 weeks presented increased colonic expression of *Tnfa*, *Il-1β*, and *Il-6* [35]. In yet another study, mice fed for a short period on a HFD presented only minor changes in colonic cytokine expression [36]. Thus, it seems that diet composition and the time window of the consumption of hypercaloric foods are important factors behind the variability found in distinct studies.

Concerning the transcripts encoding for proteins of the mucus layer, tight junctions, and adherence complexes, there were major changes, particularly in the ileum. *Muc2*, which encodes mucin 2, a secreted high-molecular-weight glycoprotein that participates in the composition of an insoluble mucus layer [37], was reduced in the non-pregnant mice on a HFD; however, it was significantly increased in pregnant females on a HFD. We found no previous studies that measured the expression of *Muc2* in the gut of pregnant mice fed a HFD; nevertheless, in a study evaluating rats fed chow, it was demonstrated that *Muc2* is an important factor against the transposition of LPS during pregnancy [38]. In addition, in pregnant mice on a HFD, there were also significant increases in the ileal expressions of *Cldn2*, *Ocln* and *Tjp1*. *Cldn2* and *Ocln* encode for claudin 2 and occludin, respectively, which are integral membrane proteins of the gut tight junctions [39,40]. We found no previous studies that evaluated the gut expression of *Cldn2* and/or *Ocln* in pregnancy as well as in pregnant mice or other rodents fed a HFD. *Tjp1* encodes tight junction protein-1, a membrane-associated guanylate kinase that plays important regulatory roles in the function of tight junctions and adherence junctions. As for *Muc2*, *Cldm2* and *Ocln*, pregnancy in mice on a HFD was accompanied by the increased expression of *Tjp1*. This is also an original finding, as no previous studies have evaluated the expression of this transcript in the gut of pregnant animals or humans. Thus, this study provides experimental evidence supporting the fact that in diet-induced obesity, pregnancy leads to a reorganization of at least three mechanisms involved in the maintenance of gut barrier integrity, which results in protection against the harmful effects of diet in promoting increased gut permeability.

As changes in gut permeability are frequently associated with changes in the gut microbiome landscape, we evaluated how pregnancy could impact this parameter. Our major findings were that in diet-induced obese mice, pregnancy was associated with an increase in *Lactobacillus murinus*, and a reduction in bacteria belonging to the *Clostridia* genus. Studies have shown that *Clostridia* are commonly increased in the gut of animal models and humans with obesity [41,42]. Particularly in women, increased *Clostridia* are associated not only with obesity, but also with other clinical components of metabolic syndrome [43]. In addition, studies have shown that weight loss is associated with reductions in gut *Clostridia* [42,44]. This reinforces the idea that the presence of large amounts of *Clostridia* in the gut may be regarded as a marker of the obesity-associated microbiome landscape. Regarding the species *Lactobacillus murinus*, a study has shown its protective role against neonatal sepsis via an attenuation of the systemic inflammatory response [45]; in addition, gut *Lactobacillus murinus* has a protective role against autoimmunity by mitigating the Th17 response [46]. Thus, our findings indicates that in obesity, pregnancy acts not by only improving the integrity of the gut barrier, but also by promoting at least two beneficial changes in the microbiome landscape.

We acknowledge that this study has some limitations: (1) For logistical reasons, we employed two distinct mice strains, Swiss and C57BL6/J (C57); nevertheless, mice were always maintained under the same housing conditions, with equal food and water quality, equal environmental humidity and temperature, equal dark/light cycles, and equal care providers. Thus, despite the genetic differences, we believe that the optimal matching in housing and care conditions contributed to a mitigation of the eventual impact on the results of this study. (2) In some experiments, the groups had different numbers of mice; this was due to losses during the execution of the protocol. Nevertheless, the statistical analysis approach was appropriate for evaluating groups of different sizes. (3) The diets had different amounts of carbohydrates; thus, although fatty acid contents account for the major differences between the diets, we cannot rule out that the overall outcomes of the intervention could also be due to the carbohydrate differences. (4) We tested a HFD with only one composition, which is rich in saturated fats; it would be also interesting to evaluate other compositions, such as diets rich in mono-, and/or polyunsaturated fats. (5) We tested only one time of exposure to the HFD; it would be interesting to evaluate if different times of exposure to the HFD, leading to different magnitudes of obesity, would impact the overall results. (6) The metabolic characterization of mice did not include parameters related to blood lipids. (7) This study is exploratory and the mechanisms behind the outcomes herein described are still unknown.

## 5. Conclusions

This is the first report showing a beneficial effect of pregnancy protecting against the harmful effects of a HFD in gut barrier integrity. The major beneficial changes occurred in the ileum and were associated with the increased expression of transcripts encoding for proteins of the mucus layer, tight junctions, and adherence complexes. Given the complex nature of the hormonal, metabolic, neurological, and behavioral changes that occur during the course of obesity, we believe the outcomes described in this study are multifactorial; however, future studies could explore, in depth, the mechanisms behind these changes, aiming at identifying potential targets for the treatment of a diet-induced loss of gut barrier integrity.

## Figures and Tables

**Table 1:** Compositions of the diets.

Component	Chow (g)	HFD (g)
Starch	437	118
Protein (casein 85%)	192	240
Dextrinized corn starch	136	70
Sucrose	100	70
Soybean oil	43	43
Lard	0	367
Fiber (cellulose)	49	49
Mineral mix (AIN-93)	30	30
Vitamin mix (AIN-93)	8	8
L-Cystine	3	3
Choline bitartrate	2	2
Total	1000	1000

**Table 2:** Real-time PCR primers used in the study.

Applied Biosystems	Assay	Ref Seq	Exon Location
*Actb*	Mm04394036_g1	AK075973.1	1–2
*Gapdh*	Mm99999915_g1	Nm_008084.2	2–3
Integrated DNA Tech	Assay	Ref Seq	Exon Location
*Cldn2*	Mm.PT.58.32319262	NM_016675(1)	1–2
*Cldn5*	Mm.PT.58.33394738.g	NM_013805(1)	1–1
*Il17a*	Mm.PT.58.6531092	NM_010552(1)	2–3
*Il6*	Mm.PT.58.13354106	NM_031168(1)	2–3
*Muc2*	Mm.PT.58.30808474.gs	XM_003945707(1)	24–25b
*Ocln*	Mm.PT.58.30118962	NM_008756(1)	6–7
*Tjp1*	Mm.PT.58.12952721	NM_001163574(2)	25–26
*Tnf*	Mm.PT.58.12575861	NM_013693(1)	2–4
*Rplp0*	Mm.PT.58.43894205	NM_007475(1)	5–6

## Data Availability

Data will be available upon request directed to the corresponding author, LAV (lavellos@unicamp.br).
